# Dying in hospital in Germany – optimising care in the dying phase: study protocol for a multi-centre bottom-up intervention on ward level

**DOI:** 10.1186/s12904-022-00960-1

**Published:** 2022-05-06

**Authors:** Kerstin Kremeike, Anneke Ullrich, Holger Schulz, Carolin Rosendahl, Kathleen Boström, Sukhvir Kaur, Nikolas Oubaid, Christina Plathe-Ignatz, Christin Leminski, Kira Hower, Holger Pfaff, Martin Hellmich, Karin Oechsle, Raymond Voltz

**Affiliations:** 1 Department of Palliative Medicine, Faculty of Medicine and University Hospital Cologne, University of Cologne, Kerpener Straße 62, 50937 Cologne, Germany; 2 Palliative Care Unit, Department of Oncology, Hematology and BMT, University Medical Center Hamburg-Eppendorf, Martinistr. 52, 20246 Hamburg, Germany; 3 Department of Medical Psychology, University Medical Center Hamburg-Eppendorf, Martinistr. 52, 20246 Hamburg, Germany; 4 Institute for Medical Sociology, Health Services Research and Rehabilitation Science, Faculty of Human Science and Faculty of Medicine, University of Cologne, Eupenerstr. 129, 50933 Cologne, Germany; 5 Institute of Medical Statistics and Computational Biology, Faculty of Medicine and University Hospital Cologne, University of Cologne, Kerpener Straße 62, 50937 Cologne, Germany; 6 Center for Integrated Oncology Aachen Bonn Cologne Duesseldorf (CIO ABCD), Faculty of Medicine and University Hospital, University of Cologne, Kerpener Straße 62, 50937 Cologne, Germany; 7 Center for Clinical Studies (ZKS), Faculty of Medicine and University Hospital, University of Cologne, Kerpener Straße 62, 50937 Cologne, Germany; 8 Center for Health Services Research (ZVFK), Faculty of Medicine and University Hospital, University of Cologne, Kerpener Straße 62, 50937 Cologne, Germany

**Keywords:** Dying phase, Dying in hospital, Bottom-up approach, University hospitals, Quality of care, Focus groups, Surveys and questionnaires, Terminal care, Attitude to death, Palliative care

## Abstract

**Background:**

Hospitals are globally an important place of care for dying people and the most frequent place of death in Germany (47%), but at the same time, the least preferred one – for both patients and their relatives. Important indicators and outcome variables indexing quality of care in the dying phase are available, and various proposals to achieve corresponding quality objectives exist. However, they are not yet sufficiently adapted to the heterogeneous needs of individual hospital wards.

**Methods:**

This multi-centre single-arm pre-post study aims at the development and implementation of context-specific measures in everyday clinical practice, followed by evaluating this approach. Therefore, (1) already existing measures regarding optimal care in the dying phase are identified applying a systematic literature review as well as an online survey and a symposium with experts. Supported by the thereby generated collection of measures, (2) a stratified sample of ten teams of different wards from two university hospitals select suitable measures and implement them in their everyday clinical practice. Circumstances of patients’ deaths on the selected wards are recorded twice, at baseline before application of the self-chosen measures and afterwards in a follow-up survey. Retrospective file analysis of deceased persons, quantitative staff surveys as well as qualitative multi-professional focus groups and interviews with relatives form the data basis of the pre-post evaluation. (3) Results are reviewed regarding their transferability to other hospitals and disseminated (inter-)nationally.

**Discussion:**

Measures that are easy to implement and appropriate to the specific situation are supposed to significantly improve the quality of care during the dying phase in hospitals and contribute to the well-being of dying patients and their relatives. Successful implementation of those measures requires consideration of the individual conditions and needs of patients and their relatives—but also of the health professionals—on the different hospital wards. Therefore, a bottom-up approach, in which the ward-specific situation is first analysed in detail and then the staff itself selects and implements measures to improve care, appears most promising for optimising care in the dying phase in hospitals.

**Trial registration:**

The study is registered in the German Clinical Trials Register (DRKS00025405).

## Background

A significant proportion of the population in developed countries dies in hospitals, making them important places of care at the end of life [1]. Even though most patients wish to die at home [1, 2], the percentage of hospital deaths ranges widely, e. g., between 34% in the Netherlands [3], 42% in Sweden [4], 47% in England [5], and 61% in Spain [3]. Although hospital care in the dying phase is often evaluated positively, there is still room for improvement—such as unmet needs for information on what to expect when someone is dying [6]. Therefore, there are considerable efforts both internationally and nationally to optimise care in the dying phase. Most famous in this regard is the *Liverpool Care Pathway* (LCP): Developed in the late 1990s [7], it was modified over the years and adapted in an international working group for application in other countries [8–10]. The LCP was designed as a ten-step palliative care approach that aims to provide consistent, high-quality, and dignified care for the dying in all settings and evolved into a complex intervention, combined with an extensive training program and audit cycles [7]. Ultimately, the proper application of the LCP failed due to this complexity [11, 12]. Nevertheless, the LCP contents are well designed and were further advanced into the international Best Care of the Dying (BCD) recommendations [13, 14]. The BCD recommendations include, e. g., recognising a patient as dying, ongoing communication with patients and their relatives, and symptom control [14]. As an alternative project to LCP/BCD, the AMBER Care Bundle (Assessment; Management; Best practice; Engagement; Recovery uncertain) was developed to deal with clinical uncertainty for hospital inpatients near the end of life [15]. This systematic treatment approach intends to support health professionals in proactively discussing the further course of action when the patient's health condition is deteriorating. In analogy to the LCP, the AMBER Care Bundle was developed "top-down" making it rather inflexible in its application [16].

In Germany, too, the hospital is the most frequent place of death (47%) [17, 18], but at the same time the least preferred one for both, patients and their relatives [19]. Annually, around 420.000 patients die in hospitals [17]. Taking into account the relatives of the deceased, an additional four to five times as many people are affected [20]. According to patients and relatives, the main reasons for dissatisfaction with dying in a hospital setting are insufficient symptom control, lack of involvement in decision-making on the further course of treatment, lack of communication between health professionals and patients, inadequate hospital environment, and interpersonal relationships with health professionals [21]. This dissatisfaction persists, although there are broadly consented targets and recommendations in Germany for best possible care in the dying phase. Within the framework of the *National Guideline on Palliative Care for Patients with Incurable Cancer*, 50 professional societies have formulated evidence- and consensus-based recommendations that should apply to all patients in the dying phase [22]. In addition, a working group of the *German Palliative Care Society (Deutsche Gesellschaft für Palliativmedizin, DGP)* elaborated actionable recommendations for the dying phase based on the BCD [13, 14]. However, their sustainable implementation and realisation has not yet taken place – particularly in non-palliative care wards, where most patients die in hospitals [23]. Outside specialised palliative care wards, health professionals often find it difficult to talk to terminally ill patients about their situation and underestimate patients’ information needs [24]. This may partly be due to the fact that death is not usually considered a possible, and in some cases acceptable outcome of treatment within the hospital setting [25]. Physicians may perceive the patients’ death as a personal failure or as a result of unsuccessful treatment. This leads to uncertainties regarding whether and how conversations about dying should be initiated – with patients and their relatives, but also within the health care team [26].

According to the *German Palliative Care Guideline*, the dying phase refers to the last three to seven days of life when, due to their health status/condition, the physical and psychological abilities of the dying patient are increasingly limited [22]. Care in the dying phase in hospitals is often intuitive and the quality depends on the motivation and competence of the responsible health professionals [27, 28]. Documentation in patient records rarely shows explicit references of actually provided end of life care [29]. Besides the primarily curative culture in acute care hospitals, increasing time and cost pressures combined with a lack of standards and routines leave little room for health professionals to reflect on care goals for patients in the dying phase [30]. However, treatment and care of those patients as well as the support of their relatives continues beyond curative options [31]. The *White Paper on Standards and Norms for Hospice and Palliative Care in Europe* [1] as well as the German *Charter for the Care of the Severely Ill and Dying* [32] emphasise the special importance of hospitals in providing individualized, best possible care for people at the end of their life and their relatives. They urge hospitals to develop and implement related recommendations and standards to this end.

Due to the COVID-19 pandemic, the quality of end of life care in hospitals gained significant additional attention and the care of dying patients under aggravated conditions has been regarded very critically. Numerous projects aim to establish good care for dying people during pandemic times [33, 34]. This emphasises the importance of profound knowledge regarding optimal care in the dying phase on acute care wards. This study aims to:

- Develop and implement context-specific measures in everyday clinical practice on non-palliative care wards using a bottom up-approach, followed by evaluating this approach
- Develop and consent a generic framework for optimising care in the dying phase in hospitals

## Methods/design

**Fig. 1:**
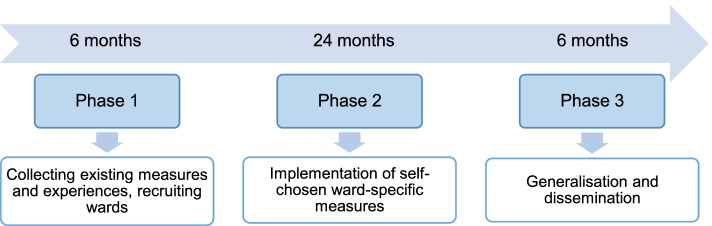
Project phases

The single-arm pre-post study to optimise care in the dying phase is located at two German university hospitals to run over a course of three years and includes three phases (Fig. 1).

### Phase 1 – Collecting existing measures and experiences; recruiting participating wards (Fig. 2)

**Fig. 2:**
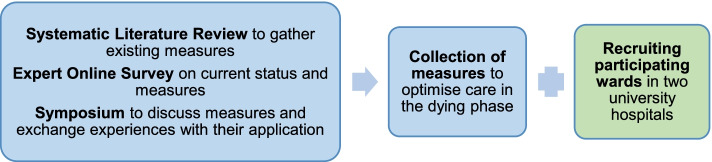
Phase 1 – Collecting existing measures and experiences; recruiting participating wards at two university hospitals

During the first phase, we gather an extensive insight on already existing measures and experiences in the care of dying patients, using a three-stage approach:

- We conduct a systematic literature review for all measures (evaluated or not) on optimisation of care in the dying phase comprising a scoping review, in which the research question is based on the SPIDER-scheme [35] with a specific search syntax and a manual search for “grey literature” using keyword search and a snowball system [35, 36]. Therewith we aim to find both established and evaluated measures as well as lean tools, relevant for the daily work in care facilities. Planned data sources are PubMed, CINAHL and Web of Science for the scoping review and Google for the grey literature search.
- To gather further information on measures to optimise care in the dying phase within the German context, a kick-off symposium with about *N* = 50 national experts in the field is held. Clinicians, researchers as well as relevant stakeholders from politics and professional societies are invited to raise the status quo of the care situation in the dying phase and to collect suitable measures for its improvement as well as to exchange experiences regarding their application. Due to Covid-19-induced travel restrictions, the symposium takes place in a hybrid format (online and on site).
- Prior to the symposium, the invited national experts are asked to take part in an online survey. Therein, besides socio-demographic data of the participants, relevant aspects regarding hospital care in the dying phase are gathered using open questions, covering the most important aspects regarding optimal care, potential barriers and facilitating factors as well as possible next steps to improve care. In order to account for the current pandemic situation, specifics due to COVID-19 are also inquired. Qualitative data from the online survey is analysed thematically [37] in order to provide a solid data basis for the symposium.

Results from the systematic literature review, the online survey and the symposium provide a broad variety of measures to optimise care in the dying phase in hospitals. *N* = 10 non-palliative wards are recruited to implement a selection of the reviewed measures or other ideas of their own in phase 2 (Fig. 2). Implementing self-chosen measures is intended to emphasize individuality and creativity and therewith to increase motivation for participation and implementation. In contrast to complex and often rigid top-down approaches [7, 15] we expect broader approval and cooperation of the hospital ward teams with this bottom-up strategy.

Both participating university hospitals have an active academic palliative medicine department and are in the catchment area of the same health insurance fund. At each of them, *n* = 5 wards are selected via purposeful sampling [38]. To capture a comprehensive picture of the conditions at the hospital wards, we aim for the greatest possible heterogeneity concerning qualification of staff, ward setting / specialty, ward size and number of deaths. Ultimately, the decisive factor for inclusion in the study is the willingness of the requested wards to participate.

### Phase 2 – Implementation of measures and evaluation of intervention

**Fig. 3:**
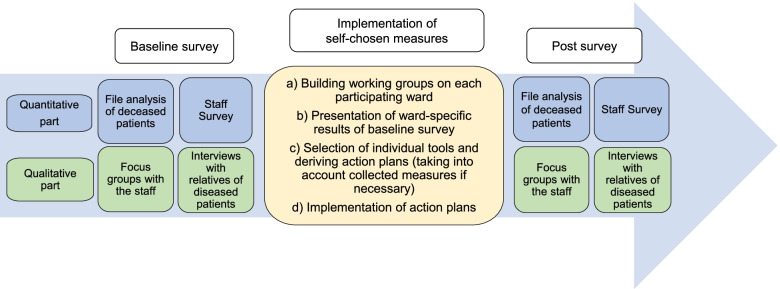
Phase 2—Implementation of self-chosen measures and pre-post-evaluation on 10 wards at two university hospitals

During phase 2, the ten different ward teams implement self-chosen measures (Fig. 3)– if desired, the measures collected in phase 1 can be presented to stimulate discussion on appropriate measures. The research team evaluates this individual bottom up-approach. The single-arm pre-post evaluation consists of a qualitative and a quantitative part to capture effects on patient care, relative support and staff experiences. We conduct data collection at all ten wards before the implementation of measures and after completion of the implementation phase. The duration of the individual implementation phases is at least six month but may vary depending on the number of deaths. The aim is to conduct the follow-up survey after 40 patients have died on each ward following the implementation.

Additionally, the research team and staff from the palliative care wards at the two university hospitals provide ongoing needs-oriented support with palliative care expertise as well as with organisational and implementation tasks in every work step at the ten wards (Fig. 3).

#### Baseline survey: Quantitative part

The quantitative part includes a retrospective file analysis of 40 deceased patients on each ward respectively before and after implementation. This yields *n* = 200 files for each hospital and *N* = 400 files in total for the baseline survey. Furthermore, a quantitative online survey is planned with *N* = 500 employees (on average *n* = 50 for each of the ten hospital wards). Included are staff members who were designated as belonging to the ward by the ward-specific contact person.

##### Retrospective patient file analysis

**Fig. 4:**
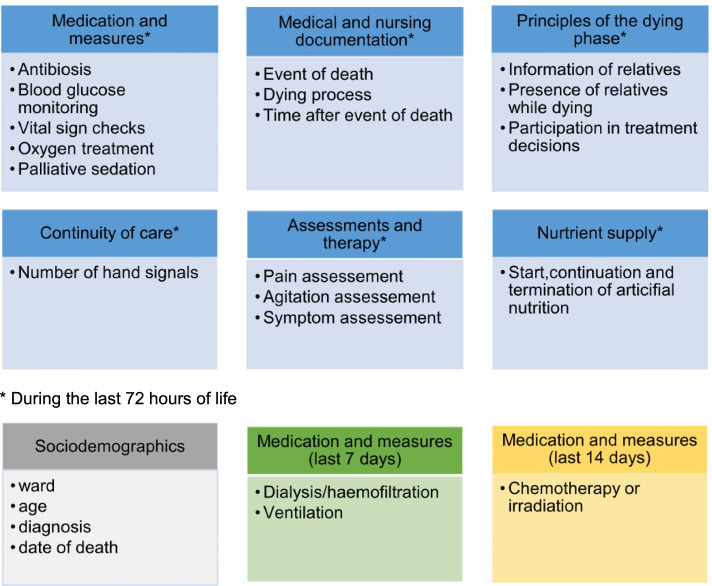
Topics of the patient file analysis [39]

An existing instrument developed in a previous project can be used for analysing the care situation of patients in the dying phase and their relatives [39]. This questionnaire is adapted to the conditions of the hospital wards participating in the present project (e.g., different documentation systems). It enables a structured retrospective analysis of routine documentation in patient files and the extent to which this reflects the contents of the key recommendations and quality indicators for care in the dying phase from the *German Palliative Care Guideline* [22]. Therewith, patient files are examined regarding available information on medication and measures, medical and nursing documentation, principles of dying phase, continuity of care, assessments and therapy, nutrient supply and sociodemographic data of the descendant patient. Most items refer to the last 72 h of the patient's life; a few items take into account the last seven or 14 days, e.g. when recording information about chemotherapy or radiation (Fig. 4).

In order to allow a differentiation of the situation before and after the outbreak of the COVID 19 pandemic within the baseline survey, 20 files of patients who died before March, 11^th^ 2020 (pandemic proclamation in Germany) and 20 files of patients who have died since then are analysed for each ward. The period for retrospective analysis before March, 11^th^ 2020 is determined by the ward with the lowest number of deaths and covers the time in which 20 patients died in this ward. For this period, either a full survey is conducted or, for wards with higher numbers of deaths, a random sample is analysed.

##### Staff survey

In an anonymous quantitative survey with an estimated *N* = 500 staff members of the participating wards, self-efficacy and feelings in dealing with dying patients and their relatives, the fear of death and dying and factors related to cooperation in the team are surveyed using validated questionnaires (see Table 1). Additionally, self-care strategies, socio-demographic data, professional background and previous experiences including satisfaction with end-of-life care in their ward are examined by a self-developed questionnaire and related stress factors [40] are also surveyed.

**Table 1:** Questionnaires used in the staff survey

Dimensions	Validated Instrument	No. of items, response options
Overall Self-Efficacy	**GES—General Self-efficacy Scale** [41]	10, 4-step Likert scale
Accompaniment of the dying and their relatives	**Self-assessment in dealing with dying patients and their relatives** [42]	25, 5-step Likert scale
Fear of death and dying	**Thanatophobia Scale** [43]	7, 7-step Likert scale
Team interaction / function	**IPS—Internal Participation Scale** [44]	6, 4-step Likert scale
**Miscellaneous questions**		
Professional self-care strategies		3, 5-step Likert scale
Sociodemographic data/ professional background and experience		11, diverse
Satisfaction with end of life care on the specific ward		1; Numeric Analog Scale
Stress factors in coping with death [40]		11, 4-step Likert

The staff of the ten participating wards receive a short information about the survey and the link to the online questionnaires via e-mail. Furthermore, postings are displayed on the wards to inform about the study and invite staff to participate in the survey. Based on experience from the earlier project [39], we assume a response rate of ≥ 50%. Reminders via e-mail are sent to all employees at regular intervals [45].

#### Baseline survey: Qualitative part

Qualitative data collection intends to ascertain subjective views on the care for dying patients and their relatives in the participating wards. We plan to conduct *N* = 20 interviews (*n* = 2 per ward) with relatives of deceased patients, each aiming for a length of about 60 min. The goal is to examine personal experiences of care and support of the interviewees to identify what is going well and potential for improvement on the respective wards. In addition, *N* = 10 multi-professional focus groups (*n* = 1 per ward; length about 90 min) with *n* = 5–10 employees each are conducted. The aim is to capture health professionals' perceptions of care and support for dying patients and their relatives. The focus groups also aim to stimulate the narrative process on the wards and are therefore themselves a kind of minimal intervention. They can show which measures are perceived as suitable among staff members.

To recruit interviewees, health professionals ask relatives who they think are suitable and, if they agree, arrange contact with the research team. The multi-professional focus groups are announced by postings on the participating wards with information about the study and asking for participation. Furthermore, ward team leaders hand out study information to their staff. Interested health professionals are invited to contact the research team.

All qualitative (group) interviews are based on semi-structured guidelines based on established models for capturing determinants of implementation (Consolidated Framework for Implementation Research (CFIR)) [46] to identify individual barriers and derive appropriate strategies to address them during tailoring [47–53]. Data collection is conducted at the place of choice of the interviewees. All (group) interviews are audio taped and analysed by means of qualitative content analysis using the qualitative data analysis software MAXQDA 2020 [54]. Content analysis is guided by the model of Miles and Huberman [55]. Findings from the qualitative surveys are pooled and conceptualized based on the CFIR, processed and made usable for tailoring ward-specific measures [48–50, 52, 53, 56].

#### Intervention

After completion of the baseline survey, the research team presents the results from the data collected on ward level to the respective staff. Additionally, the quality criteria on the dying phase as well as guideline recommendations and possible measures for their implementation including "stimulus material" on the measures compiled in phase 1 may be presented to spurring on the discussion on measures to be implemented if asked for by the ward members.

Possible measures include, e. g., the implementation of the surprise question [57] or ethical case discussions [58], but also the adaption of elements of the BCD or the AMBER Care Bundle. The presentation of the stimulus material by the research team intends to enable ward-specific working groups to independently choose and implement measures in a needs-oriented manner – individually tailored for each ward. In consultation with the ward team leaders, 3–5 staff members form a ward-specific multi-professional working group responsible for the implementation of the self-chosen measures. The ward-specific tailored measures are implemented patient-centred. The working groups are direct contacts for the research team and other ward staff and thus contribute to the smoothest possible implementation of the project. Content and results of working group meetings are documented as is information on the implementation of measures on the wards.

Over the period of the selection and implementation of measures, staff from the palliative care wards at the two university hospitals and the research team support this process continuously with medical and nursing palliative care expertise as well as in organisational and implementation tasks as much as possible.

#### Post-survey

Analogous to the baseline survey before the implementation of measures, quantitative and qualitative assessments will be repeated afterwards. This contains a retrospective patient file analysis (*N* = 400), a quantitative staff survey (*N* = 500) and focus groups with staff (*N* = 10) as well as personal interviews with relatives (*N* = 20).

For participation, the staff must have worked on the ward since implementation. Participants do not have to be identical to those who took part in the baseline survey.

On each ward, 40 cases of patient deaths taking place after implementation are aimed to be included in the post-analysis. Procedures and instruments used as well as the evaluation of the results will be identical. After intervention, the survey additionally includes the assessment of impeding and facilitating factors of implementation and its outcomes.

##### Statistical / qualitative data analysis

The pre-post comparison on the wards allows statements about the effectiveness of the implemented measures and the implementation process. The case number of (approximately) *N* = 400 patient files per phase allows detection of standardized effects of ≥ 0.2 (Cohen's *d*) with a power of ≥ 80% with two-sided type-I-error of 5% (unpaired *t* test). Statistical analysis of the data is first performed descriptively using mean ± standard deviation and percentiles (0, 25, 50, 75, 100%) for quantitative variables and absolute and relative frequencies for qualitative variables. In addition, (mixed) regression models are used for various endpoint types, stratified by wards. Missing values are described by pattern and possibly multiple imputed.

The focus of process evaluations is on implementation outcomes (acceptability, adoption, appropriateness, costs, feasibility, fidelity, penetration, sustainability) [59], associated barriers and facilitators of the implementation of the tailored ward-specific measures and the compatibility of its elements in the specific context. Information will be collected in focus groups with staff and personal interviews with relatives as part of the post survey. By means of qualitative approaches, proximal effects of interventions, procedures, practices and organizational characteristics are therefore considered and not distal outcomes of the intervention.

### Phase 3 – Generalisation and dissemination

**Fig. 5:**
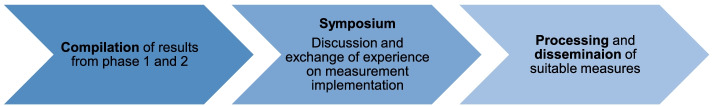
Phase 3 – Generalization and dissemination of results

After finalising the analysis and interpretation of the pre-post survey, results are summarised and contextualised for dissemination. Analogous to phase 1, a second and final symposium will be held inviting the same experts to discuss the findings obtained in the project with regard to their transferability to other hospitals or settings. The results are debated with an advisory board consisting of the nursing directors of the two university hospitals as well as representatives of the health insurance funds and (inter)national working groups on the topic. After processing the findings, materials for optimising care in the dying phase in hospitals are published on the website of the German Palliative Care Society (DGP) and continuously updated by their Working Group on the Dying Phase. Furthermore, the results are made available through (inter-)national publications and congress contributions. This will ensure both dissemination towards a scientific audience as well as easy access for clinicians who aim to use the measures in their own practice. In the long term, the indicators for optimal care in the dying phase identified as relevant in the project are also supposed to be incorporated into hospital reporting and taken into account in a seal of quality planned by the DGP for hospitals that also cultivate a palliative attitude (Fig. 5).

## Discussion

Measures that are appropriate to the specific care situation are supposed to significantly improve the quality of care during the dying phase in hospitals. We assume that even small changes on the wards have significant effects [60]. A bottom-up approach aims to increase the willingness of staff to implement respective measures [12]; the ward-specific situation is first analysed in detail and then the staff implements self-chosen measures to improve care. The early involvement of different professions and the freedom to decide which measures are to be applied specifically [61] is supposed to promote the acceptance of the project on different levels (individual, team, organization) and thus the sustainable implementation [62].

Specific issues of care during the dying phase in hospitals that have been shown since the beginning of the COVID 19 pandemic are taken into account to gain sound knowledge regarding optimal care in the dying phase even under particularly challenging circumstances.

To ensure a high quality methodological approach, an external institution will conduct the qualitative evaluation of used methods.

### Possible limitations and obstacles

Recruitment of hospital staff for surveys and to implement interventions that might cause a lasting change in care processes is known to be difficult due to lack of time, staff shortage and an already high burden on health professionals [63]. We aim to increase motivation by providing ongoing palliative care expertise of clinicians for the project as well as close contact with and organisational support of the research team. Additionally, there will be small monetary incentives for the wards [64].

In collecting data for the patient file analysis, challenges might arise due to the variations in ward documentation and associated data protection. To counter this, we train every staff member responsible for extracting data in patient files individually and in accordance with the respective documentation system. To ensure data protection and ethics regulations in each case, ward-specific solutions have to be found in order to provide anonymous information on patient data to the research team. Data analysis is conducted by the research team to ensure a high level of reliability and validity.

Recruitment of relatives of deceased patients must proceed sensitively and with caution. However, studies show that relatives of deceased patients are willing and motivated to participate in research and experience it as helpful [65].

During implementation, all wards follow specifically tailored plans with self-chosen interventions. However, due to staff working spatially close to others or on more than one ward, conversation among them is usual and promotes an unavoidable risk of information spreading. This might cause a crossover effect, which we attempt to minimize by successively starting implementation on the different wards.

Within the study, the research team has already recruited different wards in both university hospitals. The data collection for the retrospective patient file analysis and the staff survey is prepared and ready to conduct.

In summary, the project aims at implementing and evaluating specifically tailored measures to optimise hospital care in the dying phase, using a bottom-up approach. These measures might result in a generalisable, but adaptable concept that is useful in other hospital settings as well.

## Data Availability

The datasets used and/or analysed during the current study are available from the corresponding author on reasonable request.

## Abbreviations

AMBER: Assessment; Management; Best practice; Engagement; Recovery uncertain
BCD: Best Care of the Dying
DGP: Deutsche Gesellschaft für Palliativmedizin / German Palliative Care Society
GES: General Self-efficacy Scale
IPS: Internal Participation Scale
LCP: Liverpool Care Pathway
